# Disheveled binding antagonist of β-catenin 1 interacted with β-catenin and connexin 43 in human-induced pluripotent stem cells-derived cardiomyocytes

**DOI:** 10.1080/21655979.2022.2070448

**Published:** 2022-05-05

**Authors:** Jian Hou, Suiqing Huang, Yan Long, Kangni Feng, Liqun Shang, Zhuoming Zhou, Yuan Yue, Xiaolin Huang, Guangxian Chen, Zhongkai Wu

**Affiliations:** a Department of Cardiac Surgery, The First Affiliated Hospital of Sun Yat-sen University, Guangzhou, China; b Nhc Key Laboratory of Assisted Circulation, Sun Yat-sen University, Guangzhou, China

**Keywords:** DACT1, cardiomyocytes, human embryonic stem cells, induced pluripotent stem cells, β-catenin, connexin 43

## Abstract

Previously, we demonstrated that the disheveled binding antagonist of β-catenin 1 (DACT1) was involved in atrial fibrillation by regulating the reorganization of connexin 43 and β-catenin in cardiomyocytes. Little is known, however, about DACT1 in human normal myocardial cells. Therefore, we used cardiomyocytes (CMs) derived from human embryonic stem cells (ESCs) and induced pluripotent stem cells (iPSCs) to investigate the role of DACT1 and its connection with β-catenin and connexin 43. While the ESC-CMs and iPSC-CMs were differentiated using commercial differentiation kits, the cardiac-specific markers were detected by immunofluorescence. The expression level of DACT1 was detected using western blotting, whereas the interaction of DACT1 and connexin 43 or β-catenin was detected by immunofluorescence and co-immunoprecipitation (co-IP) assays. Both H1-CMs and SF-CMs were immunostained for cardiac-specific markers, including Troponin I, Troponin T, α-actinin, NKX2.5, and GATA6. While DACT1 was not expressed in both H1 ESCs and SF-iPSCs, it was, however, highly expressed in differentiated CMs, being also localized in the cytoplasm and the nucleus of differentiated CMs. Interestingly, the DACT1 expression in different nuclei was different in the same multinucleated cell. Moreover, DACT1 colocalized with β-catenin in both the cytoplasm and nucleus of differentiated CMs, and it also colocalized with connexin 43 in the perinuclear region and the gap junctions of differentiated CMs. Co-IP results showed that DACT1 could directly bind to β-catenin and connexin 43. Taken together, DACT1 interacted with β-catenin and connexin 43 in human-induced pluripotent stem cells-derived cardiomyocytes.

## Graphical abstract

**Figure:**
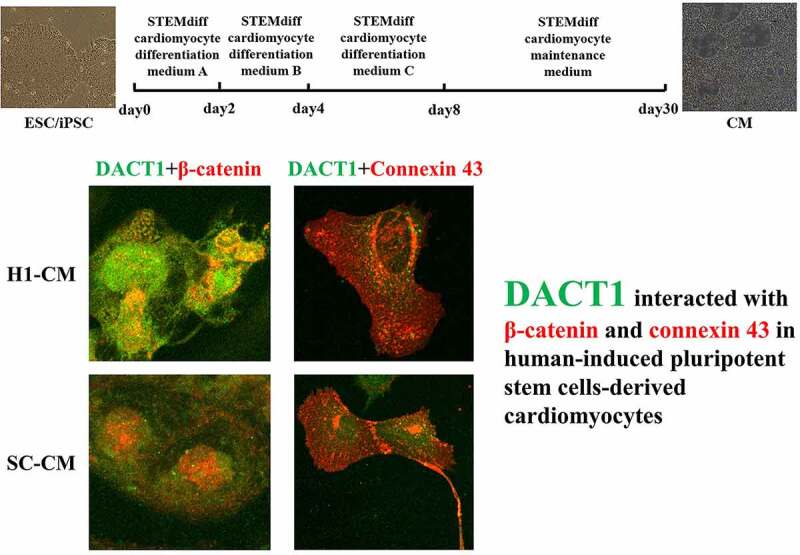

## Highlights

- DACT1 was localized in the cytoplasm and nucleus of differentiated CMs.
- DACT1 colocalized with β-catenin in the cytoplasm and nucleus of differentiated CMs.
- DACT1 co-localizes with connexin 43 in the perinuclear region and the gap junctions of CMs.

## Introduction

DACT1 (Disheveled binding antagonist of β-catenin 1), which was first found to be a Dvl-interacting protein, plays a crucial role in the development of the normal vertebrate [1] as well as human diseases [2] by affecting β-catenin, which suggests its potential role in human disease by being linked to β-catenin. Our previous study first showed that DACT1 was involved in atrial fibrillation (AF) [3], the most common cardiac arrhythmia in clinical practice, while also regulating the gap junction protein connexin 43 by accelerating cytoskeletal rearrangement via the accumulation of β-catenin in myocardial cells [3], which further suggests DACT1 might be a link between the cytoskeletal and gap junctions. To our knowledge, this is the first study to link DACT1 and connexin 43, proposing that new mechanisms mediated by DACT1 exist in cardiac disease. Tight junction-associated proteins, such as ZO-1, could bind the constituent proteins of tight junctions to the cytoskeleton, which could help maintain the stability of adhesion junctions and gap junctions [4–6]. There is, thus, an unknown mechanism between cytoskeleton and gap junctions in cardiac disease in which DACT1 might play a role, but little is known about it, especially regarding the role of DACT1 in human normal myocardial cells due to a lack of suitable research models.

Human-induced pluripotent stem cells (iPSCs) are characterized by pluripotency and a capacity for unlimited self-renewal [7]. Cardiomyocytes (CMs) derived from iPSCs or ESCs can serve as important *in vitro* models for cardiac disease or other research applications that require careful characterization of the properties of cardiomyocytes [8]. Our recent study showed that human-induced pluripotent stem cells-derived cardiomyocytes (iPSC-CM) can be obtained using a commercial cardiomyocyte differentiation kit, with a differentiation efficiency of over 95%, a mature binuclear structure, which exhibits a stable contractile activity and regular field potential rhythm, as well as excellent drug screening capabilities [9]. iPSC-CM could, therefore, be a proper cell model to investigate cardiac function and it’s *in vitro* mechanism.

Based on our previous study data, we hypothesized that DACT1 interacted with β-catenin and connexin 43 in normal cardiomyocytes, affecting the myocardial function, and to confirm this hypothesis, CMs derived from human iPSCs were used to study the role of DACT1 and its connection with β-catenin and connexin 43 in the cardiac function.

## Materials and methods

### ES cell and iPS cell culture

H1 embryonic stem cells (ESCs) were obtained from the University of Macau and had already been reported [10]. Human adult skin fibroblast-derived iPSCs (SF-iPSCs) were obtained from the South Stem Cell Bank in Guangzhou and were reported beforehand [9]. H1 ES cells and SF-iPSCs were cultured on Matrigel-coated (BD Biosciences) 6-well plates using mTeSR1 medium (StemCell Technologies), and the cells were passaged with 0.5 mM EDTA at 1:6 to 1:8 dilutions. A rock inhibitor (10 μM) was added to the mTeSR1 medium on the day of passage and removed the day after passage.

## Stem cell-derived cardiomyocyte differentiation in monolayers

While differentiation methods for cardiomyocytes have been published recently [9], cardiac-directed differentiation was initiated using STEMdiff cardiomyocyte differentiation kits when the ESCs and iPSCs reached 85%-95% confluency (day 0). On this day, the STEMdiff cardiomyocyte differentiation medium, a supplemented with Corning Matrigel, was added to the wells and cultured for two days. On day 2, the medium was removed, and the STEMdiff cardiomyocyte differentiation medium B was added to treat the cells for another two days (day 4). The cells were then changed to the STEMdiff cardiomyocyte differentiation medium C and cultured for four days (day 8). On day 8, the medium was removed and changed to complete STEMdiff cardiomyocyte maintenance medium for long-term culture. On day 30, the cells were collected for further analysis.

## Western blotting

Bearing in mind the technique that was used had already been previously described [3,9,11], the proteins were isolated from the cells with a lysis buffer (Beyotime Institute of Biotechnology, Shanghai, China) that included a protease inhibitor cocktail (Millipore, Billerica, MA, USA, 90492-K), qualified with a BCA detecting kit (Beyotime, P0012). A total 20 µg of proteins were subjected to SDS-PAGE and transferred to PVDF membranes (Millipore, Billerica, MA, USA). Primary antibodies against DACT1 (1:1000; Origene, TA809156) were used, and antigen-antibody complexes were detected by western blotting with luminol reagent (Santa Cruz Biotechnology, sc-2048). In its turn, GAPDH (Proteintech, 10494-1-AP) served as an internal reference, and at least two independent experiments were performed for each cell line. ImageJ software was used to analyze the mean light density of each band and the expression of the target genes was normalized to that of the GAPDH.

## Immunofluorescence assay

As was previously described [3,9], the technique consisted of fixing the cells were fixed with 4% paraformaldehyde for 15 min, blocked and permeabilized with 1% FBS and 0.2% Triton X-100 in PBS at room temperature for 1 hour. The cells were then incubated with primary antibody overnight at 4°C, specifically the primary antibodies against DACT1 (1:200; Origene, TA809156), Troponin I (1:200; Cell Signaling Technology, 13,083), Troponin T (1:200; Cell Signaling Technology, 5539), α-actinin (1:200; Cell Signaling Technology, 6487), NKX2.5 (1:200; Cell Signaling Technology, 8792), GATA6 (1:200; Cell Signaling Technology, 5851), β-catenin (1:200; Cell Signaling Technology, 84,805), and connexin 43 (1:200; Cell Signaling Technology, 3512) were used at the indicated dilutions. After the cells were washed three times with PBS, a secondary antibody (Alexa Fluor 488 or Alexa Fluor 568; Thermofisher, A21202 or A10042) was diluted at 1:500 and applied to the cells for 1 hour at room temperature. The cells were finally incubated with 1 μg/mL 4,6-diamidino-2-phenylindole (Thermofisher, Sigma D1306), and they were analyzed by confocal fluorescence microscopy (Zeiss 780 NLO).

## Co-immunoprecipitation assay

Bearing in mind the co-immunoprecipitation assay (co-IP) has been previously reported [12], the iPSC-CM cells were lysed with lysis buffer and centrifugation at 4°C, and then quantified by BCA assay. 5 μg of rabbit anti-DACT1 antibody (Origene, TA358350) was added into 500 μL (1 μg/μL) supernatants and then incubated overnight at 4°C. 60 μL protein A/G agarose beads (Thermo Fisher Scientific) were then added to the antigen-antibody supernatants and incubation for two hours at 4°C. After proper washing, the co-IP product was obtained with 80 μL SDS-PAGE loading buffer, and after incubation for five minutes at 100°C, 40 μL of the co-IP products was used to perform western blotting analysis using anti-β-catenin (Cell Signaling Technology, 84,805) and connexin 43 (Cell Signaling Technology, 3512)

## Results

Having previously demonstrated that DACT1 was involved in atrial fibrillation by regulating the reorganization of connexin 43 and β-catenin, little is albeit known about DACT1 in human normal myocardial cells due to a lack of suitable research models. In this study, CMs derived from human iPSCs were used to investigate the role of DACT1 and its connection with β-catenin and connexin 43 in the cardiac function.

## Generation of CMs derived by H1 ESCs and SF-iPSCs

**Figure 1.**
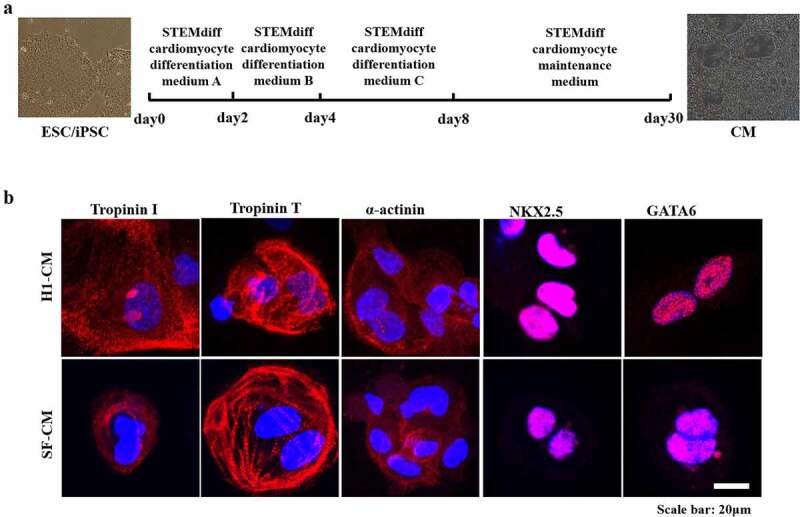
Generation of cardiomyocytes derived from iPSCs/ESCs. A. Schematic of the stepwise differentiation protocol. B. Differentiated cells were detected by immunofluorescence. Primary antibodies against cardiac-specific markers, including Troponin I, Troponin T, α-actinin, NKX2.5, and GATA6, were used. Scale bar: 20 μm.

H1 ESCs and SF-iPSCs were used to generate CMs (H1-CMs or SF-CMs) by commercial differentiation kits. While the differentiation procedure is shown in Figure 1a, the immunofluorescence results showed that both H1-CMs and SF-CMs were immunostained for cardiac-specific markers, including Troponin I, Troponin T, α-actinin, NKX2.5, and GATA6 (Figure 1b). What is more, mature evidence of binucleation was present throughout the field (Figure 1b).

## The expression pattern of DACT1 in CMs

**Figure 2.**
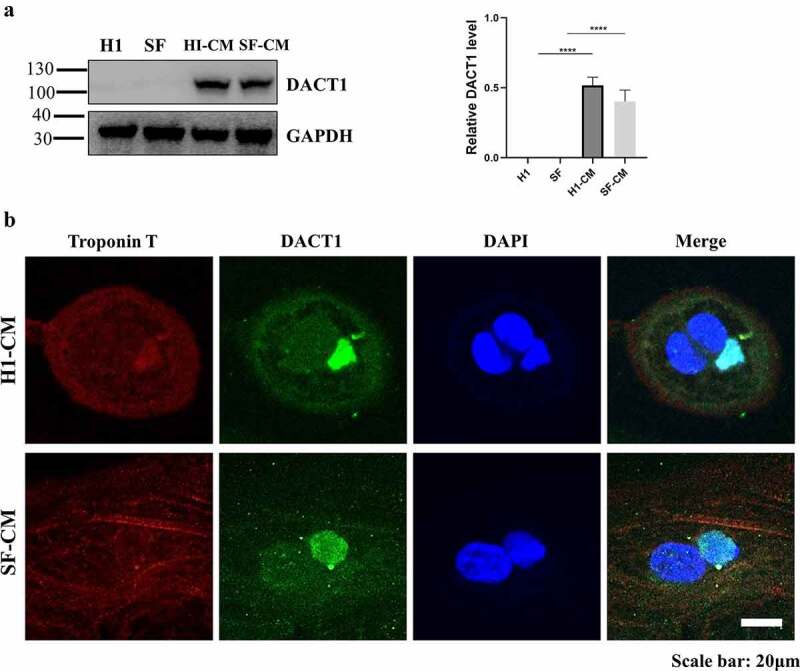
The expression pattern of DACT1 in iPSC/ESC-derived cardiomyocytes. A. The expression level of DACT1 in iPSC/ESC-derived cardiomyocytes was detected by using western blotting. The experiment was independently repeated three times and a representative graph was provided. Data are shown as the mean ± SD and comparisons of continuous variables between groups were performed with Student’s t-test. ****P < 0.00001. B. The distribution of DACT1 in iPSC/ESC-derived cardiomyocytes was detected by using immunofluorescence. Scale bar: 20 μm.

Bearing in mind that DACT1 serves as a signal transduction regulator in the cytoplasm or a transcription cofactor in the nucleus, we used western blotting and immunofluorescence assays to investigate the expression pattern of DACT1 in CMs. The former’s showed that DACT1 was not expressed in both H1 ESCs and SF-iPSCs but was highly expressed in CMs derived from H1 ESCs and SF-iPSCs (Figure 2a). Additionally, we used an immunofluorescence assay to investigate the localization of DACT1. Differentiated cells expressing Troponin T, a cardiac-specific marker, were chosen to analyze the localization of DACT1, which was localized in the cytoplasm and the nucleus of H1-CMs and SF-CMs. Interestingly, the DACT1 expression in different nuclei was different in the same multinucleated cell (Figure 2b).

## The colocalization of DACT1 with β-catenin and connexin 43

**Figure 3.**
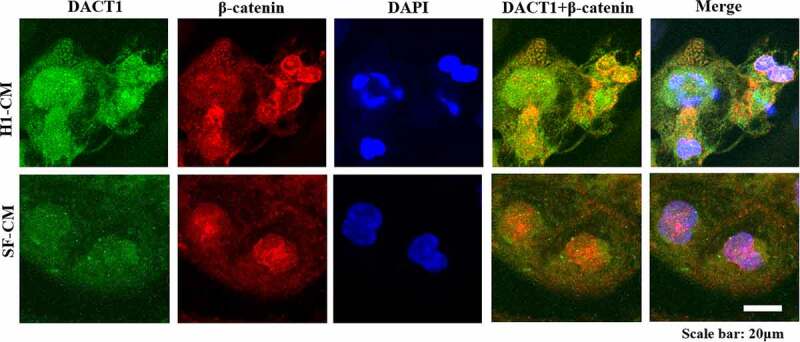
The colocalization of DACT1 and β-catenin in iPSC/ESC-derived cardiomyocytes. The colocalization of DACT1 and β-catenin in iPSC/ESC-derived cardiomyocytes was detected by using immunofluorescence. Scale bar: 20 μm.

While our previous study showed that DACT1 could regulate connexin 43 via cytoskeletal organization induced by β-catenin accumulation in cardiomyocytes of atrial fibrillation patients [3], in this study we analyzed their colocalization in CMs to further investigate their association. Not only the immunofluorescence results show that DACT1 colocalized with β-catenin in both H1-CMs and SF-CMs, but these molecules were also observed in both the cytoplasm and nucleus (Figure 3), which suggests that their functional association could exist in CMs and might play a role in the signal transduction in the cytoplasm or transcription in the nucleus.

**Figure 4.**
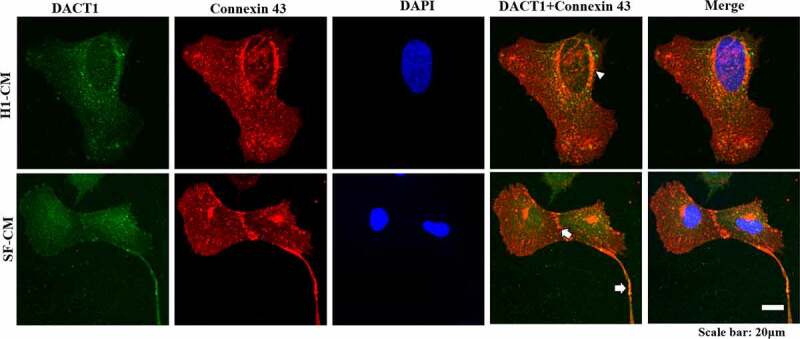
The colocalization of DACT1 and connexin 43 in iPSC/ESC-derived cardiomyocytes. The colocalization of DACT1 and connexin 43 in iPSC/ESC-derived cardiomyocytes was detected by using immunofluorescence, with the triangle indicating the perinuclear region and the arrow the gap junctions. Scale bar: 20 μm.

Moreover, the colocalization of DACT1 and connexin 43 was analyzed, and the results showed it colocalized with connexin 43 in both H1-CMs and SF-CMs, which could be observed in the perinuclear and gap junctions (Figure 4), further suggesting that DACT1 was involved in the function of connexin 43 gap junctions.

**Figure 5.**
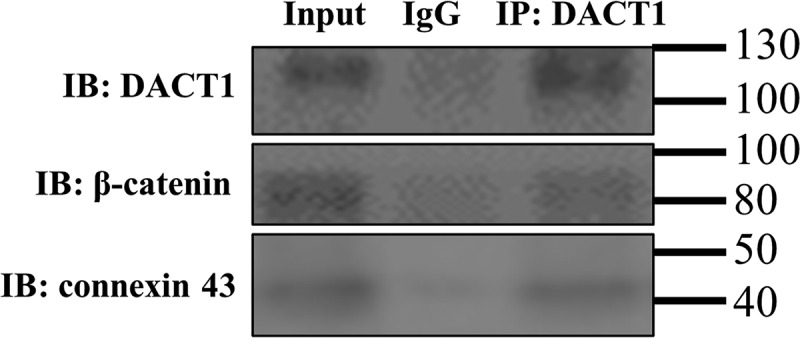
DACT1 directly interacted with β-catenin and connexin 43 in iPSC-CMs. The lysates from the iPSC-CMs were immunoprecipitated with an anti-DACT1 antibody or a nonspecific immunoglobulin G control (IgG), followed by immunoblotting with the anti-β-catenin, connexin 43, or DACT1 antibodies. The protein input served as an immunoblotting control (input), and the experiments was independently repeated twice, and a representative graph was provided.

The co-IP assay was used in the present study to investigate whether DACT1 could bind β-catenin and connexin 43 in iPSC-CMs. As shown in Figure 5, both β-catenin and connexin 43 directly interacted with DACT1 in iPSC-CMs, which suggests their direct interaction in the cardiac function.

## Discussion

DACT1, a novel bidirectional regulator of β-catenin, was found to be important in normal vertebrate development and human disease by affecting β-catenin [1–3]. DACT1 could promote or block the β-catenin degradation to regulate Wnt signaling. DACT1 could inhibit Wnt signaling by inducing β-catenin degradation complex when unphosphorylated but promoting Wnt signaling by interacting with distinct Wnt pathway binding partners when phosphorylated by CKIδ [13]. Moreover, DACT1 could induce β-catenin accumulation in the cytoplasm by inhibiting glycogen synthase kinase beta (GSK3-β) activity and interacting directly with β-catenin [14]. Nuclear β-catenin is an important transcriptional effector of the canonical Wnt signaling pathway, and cytoplasmic β-catenin is a crucial regulator of the cytoskeleton, and signal transduction [15,16]. Thus, the effect of DACT1 on β-catenin is closely related to its location.

In the present study, human H1-CMs and SF-CMs were successfully obtained using a commercial cardiomyocyte differentiation method, having this way expressed cardiac-specific markers. DACT1 was not expressed in both H1 ESCs and SF-iPSCs but was highly expressed in CMs derived from H1 ESCs and SF-iPSCs. While DACT1 was localized in the cytoplasm and nucleus of differentiated CMs, the DACT1 expression in different nuclei was interestingly different in the same multinucleated cell, while it colocalized with β-catenin in both the cytoplasm and nucleus of differentiated CMs. Additionally, DACT1 colocalized with connexin 43 in the perinuclear region and gap junctions of differentiated CMs.

Our present study showed that DACT1 colocalized with β-catenin in both the cytoplasm and nucleus of differentiated CMs, which suggests that in human iPSC-CMs and ES-CMs, DACT1 could regulate the transcriptional activity, the cytoskeleton and signal transduction in which β-catenin was involved. Interestingly, in a previous study, we had found that β-catenin was not expressed in the nucleus of the myocardium of valve disease patients, while a positive correlation with DACT1 was also observed in the cytoplasm of the myocardium [3]. It is, thus, proposed that the altered expression pattern of DACT1 and β-catenin in the myocardium may contribute to the progression of cardiac disease. Moreover, *in vivo* abnormal β-catenin expressions could lead to hypertrophic cardiomyopathy, heart failure, arrhythmias, and AF [3,17,18].

Connexin-43 is the most abundant connexin protein in the ventricular myocardium [19]. Alterations in connexin-43 expression and distribution were observed in myocardial diseases, such as hypertrophic cardiomyopathy, heart failure, AF, and ischemia [19,20]. In most cardiac disease models in which gap junctional remodeling is observed, connexin 43 is not only laterally redistributed but is also significantly reduced at the intercalated disc [21]. What is more, the reduction of connexin 43 causes gap junctional uncoupling, which leads to slowed conduction and discontinuous propagation, two key arrhythmic substrates in multiple animal models [22]. While the altered gap junction connexin 43 could be induced by actin filament reorganization [23], this could bind to ZO-1, which could bind to the C-terminus of connexin 43 and locate to the periphery of connexin 43 gap junctions, ultimately participating in regulating the speed of connexin 43 entry into the gap junction plaques and the endocytosis of gap junctions [24,25]. Our previous investigation demonstrated that DACT1 could induce F-actin filament accumulation in myocardial cells, which therefore remodeled the connexin 43 gap junction remodeled [3]. In the present study, we found that DACT1 can directly bind and colocalize with connexin 43 in the perinucleus and gap junctions of differentiated CMs, which suggests that DACT1 might be involved in the maintenance of the normal cardiac function in which connexin 43 participates.

In summary, we demonstrated that even though DACT1 was not expressed in both H1 ESCs and SF-iPSCs, it was highly expressed in CMs derived from H1 ESCs and SF-iPSCs. Additionally, DACT1 was localized in the cytoplasm and nucleus of differentiated CMs, and it was also colocalized and directly interacted with β-catenin and connexin 43 in differentiated CMs. Finally, DACT1 interacted with β-catenin and connexin 43 in human-induced pluripotent stem cells-derived cardiomyocytes.

## Conclusion

DACT1 was not expressed in both H1 ESCs and SF-iPSCs but was highly expressed in ESCs-CMs and iPSCs-CMs, which was detected in the cytoplasm and the nucleus of differentiated CMs. Additionally, it was colocalized and directly interacted with β-catenin and connexin 43 in differentiated CMs and human-induced pluripotent stem cells-derived cardiomyocytes.

## References

[1] Cheyette BNR, Waxman JS, Miller JR, et al. Dapper, a dishevelled-associated antagonist of β-Catenin and JNK signaling, is required for notochord formation. Dev Cell. 2002;2(4):449–461. 11970895 10.1016/s1534-5807(02)00140-5

[2] Hou J, Li E-M, Shen J-H, et al. Cytoplasmic HDPR1 is involved in regional lymph node metastasis and tumor development via beta-catenin accumulation in esophageal squamous cell carcinoma. J Histochem Cytochem. 2011;59(7):711–718. 21525190 10.1369/0022155411409941 PMC3201161

[3] Hou J, Yue Y, Hu B, et al. DACT1 involvement in the cytoskeletal arrangement of cardiomyocytes in atrial fibrillation by regulating Cx43. Braz J Cardiovasc Surg. 2019;34:711–722. 31545578 10.21470/1678-9741-2019-0033 PMC6894021

[4] Stevenson BR, Siliciano JD, Mooseker MS, et al. Identification of ZO-1: a high molecular weight polypeptide associated with the tight junction (zonula occludens) in a variety of epithelia. J Cell Biol. 1986;103:755–766. 3528172 10.1083/jcb.103.3.755 PMC2114282

[5] Fanning AS, Anderson JM. Zonula occludens-1 and −2 are cytosolic scaffolds that regulate the assembly of cellular junctions. Ann N Y Acad Sci. 2009;1165:113–120. 19538295 10.1111/j.1749-6632.2009.04440.x PMC3759978

[6] Toyofuku T, Yabuki M, Otsu K, et al. Direct association of the gap junction protein connexin-43 with ZO-1 in cardiac myocytes. J Biol Chem. 1998;273:12725–12731. 9582296 10.1074/jbc.273.21.12725

[7] Takahashi K, Tanabe K, Ohnuki M, et al. Induction of pluripotent stem cells from adult human fibroblasts by defined factors. Cell. 2007;131:861–872. 18035408 10.1016/j.cell.2007.11.019

[8] Zhang J, Wilson GF, Soerens AG, et al. Functional cardiomyocytes derived from human induced pluripotent stem cells. Circ Res. 2009;104:e30–41. 19213953 10.1161/CIRCRESAHA.108.192237 PMC2741334

[9] Long Y, Hou J, Tang F, et al. Proarrhythmic effects induced by benzethonium chloride and domiphen bromide in vitro and in vivo. Toxicol Appl Pharmacol. 2021;431:115731. 34592322 10.1016/j.taap.2021.115731

[10] Yang Y, Ren Z, Xu F, et al. Endogenous IGF signaling directs heterogeneous mesoderm differentiation in human embryonic stem cells. Cell Rep. 2019;29:3374–3384 e3375. 31825822 10.1016/j.celrep.2019.11.047

[11] Huang X, Hou J, Huang S, et al. Melatonin ameliorates myocardial injury by reducing apoptosis and autophagy of cardiomyocytes in a rat cardiopulmonary bypass model. PeerJ. 2021;9:e11264. 33954056 10.7717/peerj.11264 PMC8053380

[12] Wang L, Yue Y, Yang X, et al. Platelet derived growth factor alpha (PDGFRalpha) induces the activation of cardiac fibroblasts by activating c-Kit. Med Sci Monit. 2017;23:3808–3816. 28780584 10.12659/MSM.906038 PMC5555739

[13] Teran E, Branscomb AD, Seeling JM. Dpr Acts as a molecular switch, inhibiting Wnt signaling when unphosphorylated, but promoting Wnt signaling when phosphorylated by casein kinase Idelta/epsilon. PLoS One. 2009;4:e5522. 19440376 10.1371/journal.pone.0005522 PMC2679210

[14] Yuan G, Wang C, Ma C, et al. Oncogenic function of DACT1 in colon cancer through the regulation of beta-catenin. PLoS One. 2012;7:e34004. 22470507 10.1371/journal.pone.0034004 PMC3309901

[15] Gu S, Honisch S, Kounenidakis M, et al. Membrane androgen receptor down-regulates c-src-activity and beta-catenin transcription and triggers GSK-3beta-phosphorylation in colon tumor cells. Cell Physiol Biochem. 2014;34:1402–1412. 25301365 10.1159/000366346

[16] Han X, Wang DZ, Yuan M, et al. Lemur tyrosine kinase 2 silencing inhibits the proliferation of gastric cancer cells by regulating GSK-3beta phosphorylation and beta-catenin nuclear translocation. Bioengineered. 2022;13:6231–6243. 34719320 10.1080/21655979.2021.1999375 PMC8982461

[17] Masuelli L, Bei R, Sacchetti P, et al. Beta-catenin accumulates in intercalated disks of hypertrophic cardiomyopathic hearts. Cardiovasc Res. 2003;60:376–387. 14613867 10.1016/j.cardiores.2003.08.005

[18] Swope D, Cheng L, Gao E, et al. Loss of cadherin-binding proteins beta-catenin and plakoglobin in the heart leads to gap junction remodeling and arrhythmogenesis. Mol Cell Biol. 2012;32:1056–1067. 22252313 10.1128/MCB.06188-11 PMC3295003

[19] Michela P, Velia V, Aldo P, et al. Role of connexin 43 in cardiovascular diseases. Eur J Pharmacol. 2015;768:71–76. 26499977 10.1016/j.ejphar.2015.10.030

[20] Luo MH, Li YS, Yang KP. Fibrosis of collagen I and remodeling of connexin 43 in atrial myocardium of patients with atrial fibrillation. Cardiology. 2007;107:248–253. 16953110 10.1159/000095501

[21] Fontes MS, van Veen TA, de Bakker JM, et al. Functional consequences of abnormal Cx43 expression in the heart. Biochim Biophys Acta. 2012;1818:2020–2029. 21839722 10.1016/j.bbamem.2011.07.039

[22] Kleber AG, Saffitz JE. Role of the intercalated disc in cardiac propagation and arrhythmogenesis. Front Physiol. 2014;5:404. 25368581 10.3389/fphys.2014.00404 PMC4201087

[23] Li J. Alterations in cell adhesion proteins and cardiomyopathy. World J Cardiol. 2014;6:304–313. 24944760 10.4330/wjc.v6.i5.304 PMC4062122

[24] Giepmans BN, Moolenaar WH. The gap junction protein connexin43 interacts with the second PDZ domain of the zona occludens-1 protein. Curr Biol. 1998;8:931–934. 9707407 10.1016/s0960-9822(07)00375-2

[25] Rhett JM, Jourdan J, Gourdie RG. Connexin 43 connexon to gap junction transition is regulated by zonula occludens-1. Mol Biol Cell. 2011;22:1516–1528. 21411628 10.1091/mbc.E10-06-0548 PMC3084674

